# α-tomatine inhibits growth and induces apoptosis in HL-60 human myeloid leukemia cells

**DOI:** 10.3892/mmr.2015.3238

**Published:** 2015-01-22

**Authors:** HUARONG HUANG, SHAOHUA CHEN, JEREMIAH VAN DOREN, DONGLI LI, CHELSEA FARICHON, YAN HE, QIUYAN ZHANG, KUN ZHANG, ALLAN H CONNEY, SUSAN GOODIN, ZHIYUN DU, XI ZHENG

**Affiliations:** 1 Allan H. Conney Laboratory for Anticancer Research, Guangdong University of Technology, Guangzhou, Guangdong 510006, P.R. China; 2 Susan Lehman Cullman Laboratory for Cancer Research, Department of Chemical Biology, Ernest Mario School of Pharmacy, Rutgers, The State University of New Jersey, Piscataway, NJ 08854, USA; 3 Department of Otolaryngology, Guangdong Provincial People’s Hospital, Guangzhou, Guangdong 510006, P.R. China; 4 Division of Medical Oncology, Rutgers Cancer Institute of New Jersey, New Brunswick, NJ 08903, USA

**Keywords:** glycoalkaloid, α-tomatine, leukemia, apoptosis, growth inhibition, cholesterol

## Abstract

α-tomatine is a glycoalkaloid that occurs naturally in tomatoes (*Lycopersicon esculentum*). In the present study, the effects of α-tomatine on human myeloid leukemia HL-60 cells were investigated. Treatment of HL-60 cells with α-tomatine resulted in growth inhibition and apoptosis in a concentration-dependent manner. Tomatidine, the aglycone of tomatine had little effect on the growth and apoptosis of HL-60 cells. Growth inhibition and apoptosis induced by α-tomatine in HL-60 cells was partially abrogated by addition of cholesterol indicating that interactions between α-tomatine and cell membrane-associated cholesterol may be important in mediating the effect of α-tomatine. Activation of nuclear factor-κB by the phorbol ester, 12-O-tetradecanoylphorbol-13-acetate failed to prevent apoptosis in HL-60 cells treated with α-tomatine. In animal experiments, it was found that treatment of mice with α-tomatine inhibited the growth of HL-60 xenografts *in vivo*. Results from the present study indicated that α-tomatine may have useful anti-leukemia activities.

## Introduction

**Figure 1:**
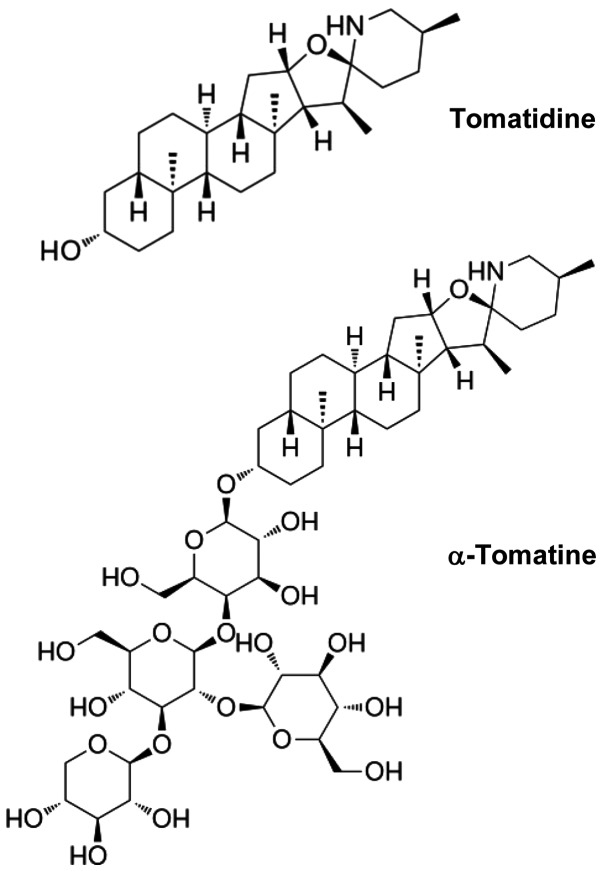
Structures of α-tomatine and tomatidine.

The solanum-steroid-alkaloids found in plants of the *Solanum* species are of interest as a starting material for the synthesis of steroid hormones and exhibit notable pharmaceutical and toxicological properties (1,2). α-tomatine and tomatidine (Fig. 1), occur naturally in tomatoes (*Lycopersicon esculentum*), belonging to the group of solanum-steroid-alkaloids. α-tomatine is a glycoalkaloid consisting of an aglycone moiety (tomatidine) and a tetrasaccharide moiety (β-lycotetraose), which is composed of two molecules of glucose, one galactose and one xylose; the four monosaccharides form a branched structure, which is attached at the C-3 position of the aglycone (Fig. 1). Although it was known that an enzyme, termed tomatinase, catalyzes the hydrolysis of tomatine into tomatidine and β-lycotetraose (3,4), the biosynthesis and metabolism of tomatine and tomatidine remain to be elucidated. Unripe green tomatoes may contain up to 500 mg/kg tomatine fresh fruit weight. The compound is partly degraded as the tomato ripens until at maturity, levels in red tomatoes are ~5 mg/kg fresh fruit weight (5). α-tomatine has also been found at high concentrations in leaves, stems and roots, suggesting that it may be important in resistance to potential pathogens (3,4).

α-tomatine has been reported to exert toxicity against certain types of microorganisms (6–8). Previous studies have also demonstrated that α-tomatine has cytotoxic effects on insect and rat cells (9–11). In previous years, the anticancer effect of α-tomatine has been investigated. α-tomatine inhibits the growth of human cancer cells, including the HT-29 colon cancer, HepG2 liver cancer, A549 lung cancer, PC-3 prostate cancer and MCF-7 breast cancer cell lines (12–15). α-tomatine also inhibits the growth of lymphoma and leukemia cells (16,17). Although α-tomatine has been revealed to have anticancer activities in different cancer cells, the mechanisms of action and particularly the primary target(s) remain to be elucidated. In the present study, the effects and mechanisms of α-tomatine were examined in HL-60 human myeloid leukemia cells, which are widely used as a model system to investigate the effect of different anticancer agents (18,19). The present study found that α-tomatine markedly inhibited growth and induced apoptosis in HL-60 cells. α-tomatine also inhibited the *in vivo* growth of HL-60 cells in a mouse xenograft model.

## Materials and methods

### Cells and reagents

HL-60 cells were obtained from the American Type Culture Collection (Rockville, MD, USA). Tomatidine, α-tomatine and cholesterol were purchased from Sigma-Aldrich (St. Louis, MO, USA). RPMI-1640, penicillin-streptomycin, L-glutamine and fetal bovine serum (FBS) were purchased from Gibco-BRL (Grand Island, NY, USA). HL-60 cells were maintained in RPMI-1640 culture medium containing 10% FBS that was supplemented with penicillin (100 U/ml), streptomycin (100 *μ*g/ml) and L-glutamine (300 *μ*g/ml) (Gibco-BRL). Cultured cells were grown at 37°C in a humidified atmosphere of 5% CO_2_ and were passaged twice a week.

### Determination of viable cells

Cell viability was determined by the trypan blue exclusion assay, as described previously (20), which was performed by mixing 80 *μ*l of cell suspension and 20 *μ*l of 0.4% trypan blue solution for 2 min. Blue cells were counted as dead cells and the cells that did not absorb dye were counted as live cells.

### Morphological assessment of apoptotic cells

Apoptosis was determined by morphological assessment in cells stained with propidium iodide (21). Briefly, cytospin slides were prepared following each experiment and cells were fixed with acetone/methanol (1:1) for 10 min at room temperature, followed by 10 min with propidium iodide staining (1 *μ*g/ml in phosphate-buffered saline; Gibco-BRL) and analyzed using a fluorescence microscope (Nikon Eclipse TE200; Nikon, Tokyo, Japan). Apoptotic cells were identified by classical morphological features, including nuclear condensation, cell shrinkage and formation of apoptotic bodies (21).

### Nuclear extract preparation and electrophoretic mobility shift assay (EMSA)

Mini-extracts prepared from cells (1×10^7^ cells/ml) were collected by centrifugation (13000 × g for 10 min), resuspended in hypotonic buffer and incubated on ice to obtain the nuclear pellet as described in our previous study (22). Oligonucleotides were synthesized by the DNA Synthesis and Sequencing Laboratory at the Cancer Institute of New Jersey (New Brunswick, NJ, USA). Double-stranded oligonucleotides were labeled by incubation with the Klenow enzyme fragment of DNA polymerase in the presence of ^32^P-dCTP, ^32^P-dGTP, dATP and dTTP deoxynucleoside triphosphates. Radiolabeled oligonucleotides (at least 1×10^8^ cpm/*μ*g) were incubated with 8 *μ*g of nuclear protein and 3 *μ*g of poly(dI-dC) in a total volume of 16 *μ*l. DNA-protein complexes were analyzed by electrophoresis on 6% acrylamide gels run in 1X Tris-borate buffer (Sigma-Aldrich) (22).

### HL-60 xengrafts in immunodeficient mice

Female severe combined immunodeficient (SCID) mice (6–7 weeks old) were obtained from Taconic Farms Inc. (Germantown, NY, USA). The animals were housed in sterile filter-capped microisolator cages and provided with sterilized food and water. HL-60 cells (1.0×10^6^ cells/0.1 ml/mouse) suspended in 50% Matrigel (Collaborative Research, Bedford, MA, USA) in RPMI-1640 medium were injected subcutaneously into the right flank of the mice. After 3 weeks, mice with subcutaneous tumors were divided into two groups. One group of animals received intraperitoneal (IP) injection of vehicle, which consisted of propylene glycol, polysorbate 80, benzyl alcohol, ethanol and water (40: 0.5: 1: 10: 48.5). The other group of animals received IP injection of α-tomatine (5 mg/kg; 5 *μ*l vehicle/g). The mice received treatment three times a week for 3 weeks. Tumor size (length × width) and body weight were measured. The animal experiment was performed under an Institutional Animal Care and Use Committee-approved protocol (#02-001; Rutgers University, Piscataway, NJ, USA).

### Statistical analysis

The analysis of variance method with the Tukey-Kramer test was used for the comparison of growth inhibition and apoptosis. Student’s t-test was used to assess the differences of tumor size and body weight between the control group and the treatment group.

## Results

### Effects of α-tomatine on the growth of HL-60 cells

**Figure 2:**
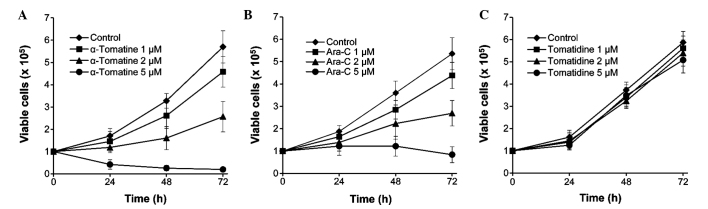
Effects of α-tomatine, tomatidine and Ara-C on the growth of HL-60 cells. HL-60 cells were seeded at a density of 1×10^5^ cells/ml in 35 mm tissue culture dishes and treated with various concentrations of (A) α-tomatine, (B) Ara-C and (C) tomatidine for 24, 48 and 72 h. The number of viable cells was determined using the trypan blue exclusion assay. Each value represents the mean ± standard error of the mean from three separate experiments. Ara-c, cytosine arabinoside.

HL-60 cells were treated with different concentrations of α-tomatine for 72 h. The number of viable cells was determined at 24, 48 and 72 h using the trypan blue exclusion assay (Fig. 2). Treatment of HL-60 cells with α-tomatine resulted in a time- and concentration-dependent growth inhibition. α-tomatine at 2 and 5 *μ*M markedly inhibited the growth of HL-60 cells. Treatment with α-tomatine (5 *μ*M) resulted in ~98% decrease in the number of viable cells (Fig. 2A). Cytosine arabinoside (Ara-C), a commonly used chemotherapeutic drug for the treatment of myeloid leukemia, was included in the experiment. Treatment of HL-60 cells with Arc-C also resulted in growth inhibition in a time- and concentration-dependent manner (Fig. 2B). α-tomatine at 5 *μ*M exhibited a more significant growth inhibitory effect than Ara-C. By contrast to the marked growth inhibitory effect of α-tomatine, tomatidine (the aglycone of tomatine, see Fig. 1) had little or no effect on the growth of HL-60 cells (Fig. 2C).

### Effect of α-tomatine on the apoptosis of HL-60 cells

**Figure 3:**
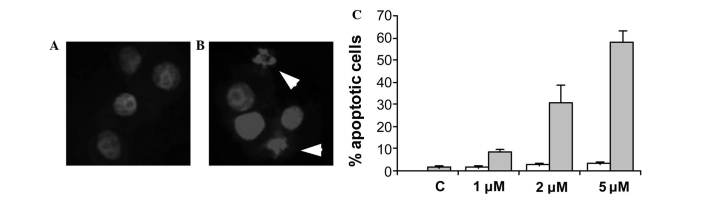
Effect of α-tomatine and tomatidine on the apoptosis of HL-60 cells. HL-60 cells were seeded at a density of 1×10^5^ cells/ml in 35 mm tissue culture dishes and treated with various concentrations of α-tomatine and tomatidine for 48 h. Apoptosis was determined by propidium iodide staining and morphological assessment. Representative micrographs of propidium iodide-stained cells from the (A) control group and cells treated with (B) 2 *μ*M α-tomatine (arrows indicate apoptotic cells). (C) Percentage of apoptotic cells as determined by morphological assessment in HL-60 cells treated with different concentrations of α-tomatine (grey bar) and tomatidine (open bar). Each value represents the mean ± standard error of the mean from three separate experiments.

The effects of α-tomatine and tomatidine on stimulation of apoptosis in HL-60 cells were determined using morphological assessment of apoptotic cells. Apoptotic cells were identified by classical morphological features, including nuclear condensation, cell shrinkage and formation of apoptotic bodies. Morphologically distinct apoptotic cells from representative samples are shown in Fig. 3B. Treatment of HL-60 cells with α-tomatine stimulated apoptosis in a concentration-dependent manner (Fig. 3C). A low concentration of α-tomatine (1 *μ*M) had little stimulatory effect on HL-60 cell apoptosis. Treatment with 2 *μ*M α-tomatine resulted in ~30% apoptotic cells and treatment with a higher concentration of α-tomatine (5 *μ*M) resulted in ~60% apoptotic cells. By contrast, tomatidine had little or no stimulatory effect on the apoptosis of HL-60 cells (Fig. 3C).

### Effects of cholesterol on α-tomatine-induced growth inhibition and apoptosis

**Figure 4:**
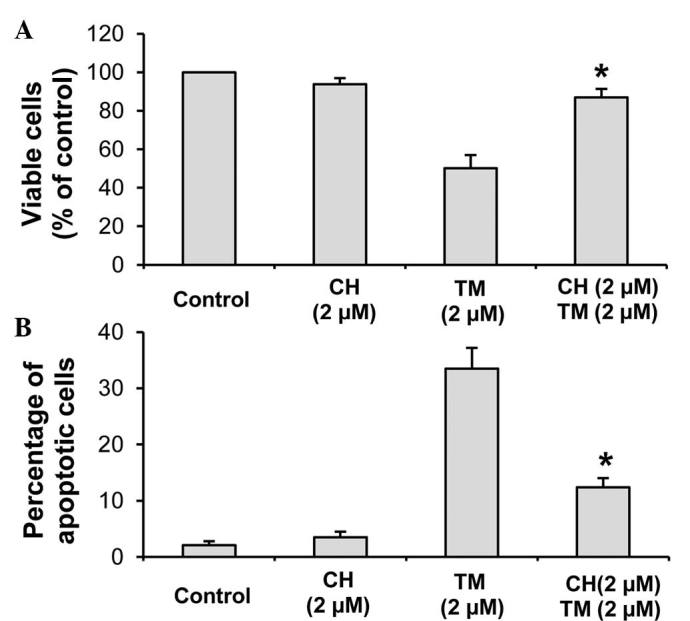
Effects of CH on TM-induced growth inhibition and apoptosis. HL-60 cells were seeded at a density of 1×10^5^ cells/ml in 35 mm tissue culture dishes and treated with TM in the presence or absence of CH for 48 h. (A) The number of viable cells was determined by the trypan blue exclusion assay. (B) Apoptosis was determined by propidium iodide staining and morphological assessment. Each value represents the mean ± standard error of the mean from three separate experiments. Differences in the number of viable and apoptotic cells between the TM-treated group and TM+CH-treated group were analyzed by analysis of variance with the Tukey-Kramer multiple comparison test. ^*^P<0.01 vs. TM-treated group. CH, cholesterol; TM, α-tomatine.

It has been established that α-tomatine and tomatidine can form complexes with cholesterol (23,24). It was further investigated whether binding of α-tomatine with cell membrane cholesterol is required for the effect of this compound on growth inhibition and apoptosis in HL-60 cells. In these experiments, HL-60 cells were treated with α-tomatine in the presence or absence of cholesterol and cell growth and apoptosis were determined. The present study found that the addition of cholesterol significantly abrogated α-tomatine-induced growth inhibition and apoptosis in HL-60 cells. As shown in Fig. 4A, treatment with 2 *μ*M α-tomatine decreased the number of viable cells by ~50%. The addition of an equal molar concentration of cholesterol abrogated the effect of α-tomatine. The addition of an equal molar concentration of cholesterol also abrogated the effect of tomatine on the stimulation of apoptosis in HL-60 cells (Fig. 4B).

### Effects of TPA on α-tomatine-induced growth inhibition and apoptosis

**Figure 5:**
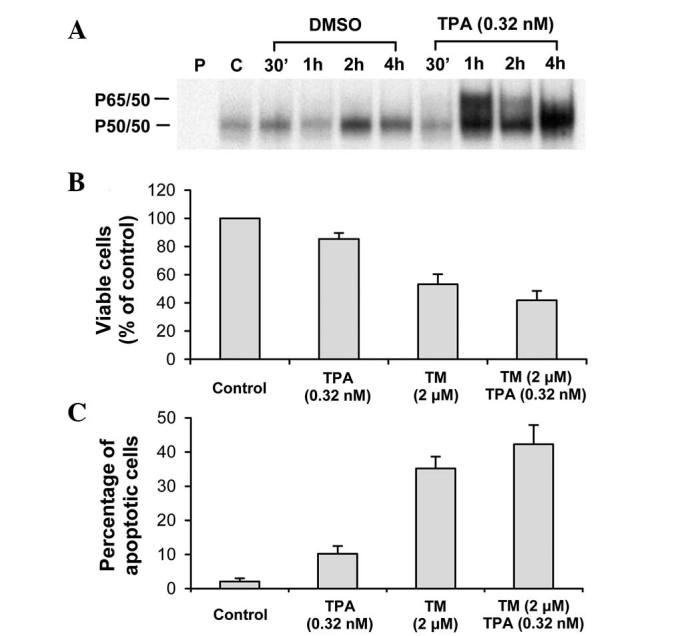
Effects of pre-treatment with TPA on TM-induced apoptosis in HL-60 cells. (A) HL-60 cells were seeded in 100 mm culture dishes and treated with 0.32 nM TPA for different time intervals. Activation of NF-κB in HL-60 cells was determined by electrophoretic mobility shift assay. P=probe only. C=control. Treatment of HL-60 cells with TPA for 1 h resulted in a marked activation of NF-κB. (B and C) HL-60 cells were seeded at a density of 1×10^5^ cells/ml in 35 mm culture dishes. The cells were treated with TM for 48 h with or without pre-treatment with 0.32 nM TPA for 1 h. (B) The number of viable cells was determined by the trypan blue exclusion assay and expressed as a percentage of the control. (C) Apoptosis was determined by propidium iodide staining and morphological assessment. TPA, 12-O-tetradecanoylphorbol-13-acetate; TM, α-tomatine; CH, cholesterol; DMSO, dimethyl sulfoxide.

The phorbol ester TPA was demonstrated to activate the nuclear transcription factor, nuclear factor (NF)-κB in cancer cells, including leukemia cells (22,25,26). Multiple studies have indicated that NF-κB has an anti-apoptotic effect and activation of NF-κB protects cancer cells, including leukemia cells from apoptosis (27–29). It is therefore important to investigate whether pretreatment with TPA may protect HL-60 cells from α-tomatine-induced apoptosis. In initial experiments, HL-60 cells were treated with TPA and its effect on activation of NF-κB was determined using the EMSA. As shown in Fig. 5A, treatment of HL-60 cells with TPA for 1 h caused a marked activation of NF-κB. In subsequent experiments, HL-60 cells were pretreated with TPA for 1 h and then treated with tomatine. However, pretreatment with TPA failed to protect the cells from α-tomatine-induced growth inhibition (Fig. 5B) and apoptosis (Fig. 5C).

### Effect of α-tomatine on the growth of HL-60 xenograft tumors

**Figure 6:**
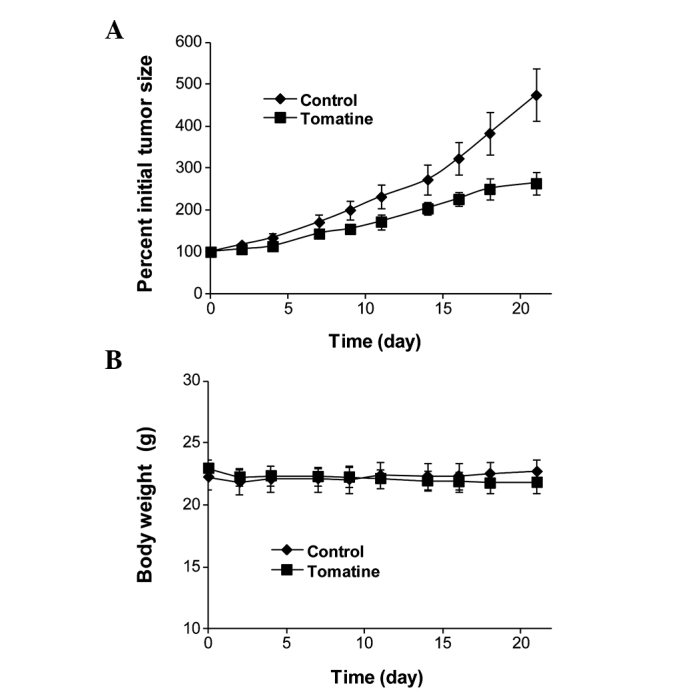
Effects of α-tomatine on (A) the growth of HL-60 xenografts and (B) body weight of SCID mice. Female SCID mice were injected subcutaneously with HL-60 cells in 50% Matrigel (1.0×10^6^ cells/0.1 ml) suspended in RPMI medium. After 3 weeks, mice with HL-60 xenograft tumors (0.6–1.0 cm wide and 0.6–1.0 cm long) were intraperitoneally injected with vehicle (5 *μ*l/g body weight; n=12) or with α-tomatine (5 mg/kg body weight; n=12) three times a week for 3 weeks. (A) Tumor size (length x width; cm^2^) was measured and expressed as a percentage of initial tumor size. (B) Body weight (g). SCID, severe combined immunodeficient.

Female SCID mice with subcutaneous HL-60 xenograft tumors were injected IP with vehicle (5 *μ*l/g body weight) or α-tomatine (5 mg/kg; 5 *μ*l vehicle/g) three times a week for 3 weeks. As shown in Fig. 6, treatment with α-tomatine significantly inhibited the growth of HL-60 tumors. The mean ± standard error of the mean (SEM) for the percentage of the initial tumor size after 3 weeks of treatment was 475.2±62.2 for the control group and 263.9±25.7 for the α-tomatine-treated group. Statistical analysis using Student’s t-test revealed that the difference in the percentage of the initial tumor size between the control group and the α-tomatine-treated group were statistically significant (P<0.01). The mean ± SEM for the body weight (g) was 22.7±0.9 for the vehicle-treated control group and 21.8±1.0 for the α-tomatine-treated group. Statistical analysis using Student’s t-test revealed that the difference in the body weight between the control group and the treatment group was not statistically significant (P>0.05).

## Discussion

Glycoalkaloids are nitrogen-containing secondary plant metabolites found in numerous plants, including potatoes and tomatoes (30). The glycoalkaloid α-tomatine has been hypothesized to be involved in host-plant resistance against phytopathogens and has a variety of pharmacological and toxicological properties in animals and humans (5). α-tomatine has previously been found to exert anticancer activities (13–17,31). In the present study, the effect of α-tomatine and its aglycone, tomatidine was determined in myeloid leukemia HL-60 cells. Although it has previously been reported that α-tomatine inhibited growth and induced apoptosis in leukemia cells, the effect of tomatidine on leukemia cells has not been investigated. In the present study it was demonstrated for the first time, to the best of our knowledge, that α-tomatine, but not tomatidine inhibited growth and induced apoptosis in HL-60 cells indicating the importance of the glycone compartment of this compound. It was also found that α-tomatine was as potent as the widely used anti-leukemia drug Ara-C for inhibiting the growth and stimulating the apoptosis of HL-60 cells.

The mechanisms by which α-tomatine induces growth inhibition and apoptosis in myeloid leukemia cells remain to be elucidated. A previous study revealed that treatment with α-tomatine resulted in activation of Bak and Mcl-1s, and caused release of apoptosis inducing factor and suppression of survivin (16). Other studies demonstrated that α-tomatine inhibited the NF-κB and phosphatidyl-inositol-3-kinase/Akt signaling pathways activation in lung and prostate cancer cells (13,15). However, the primary cellular target(s) for α-tomatine and its mechanisms for modulating apoptosis-associated pathways remain to be elucidated. Previous *in vitro* studies revealed that α-tomatine forms a robust complex with cholesterol in aqueous media (23,24) and α-tomatine was used as a cholesterol probe (32). Keukens *et al* (33) demonstrated that α-tomatine and other glycoalkaloids interacted with membrane-associated cholesterol. In the present study, it was investigated whether the interaction of α-tomatine with cell membrane-associated cholesterol is essential for its effects on leukemia cells. It was found that inhibiting the interaction of α-tomatine with cell membrane-associated cholesterol by addition of equal molar concentrations of free cholesterol in the medium abrogated the effect of α-tomatine. This is the first study, to the best of our knowledge, indicating that the interaction of tomatine with cholesterol is important for mediating its effect on growth and apoptosis in leukemia cells. The interaction of α-tomatine with cholesterol and the disruptive effect on the cell membrane may be one of the mechanisms by which α-tomatine induces apoptosis in HL-60 cells. It is also possible that the formation of complexes of α-tomatine and cholesterol may modulate the responsiveness of cell membrane receptors to growth stimuli and thus decrease the growth of HL-60 cells.

To further investigate whether the glycone group is required for α-tomatine to inhibit growth and induce apoptosis, the effects of α-tomatine with its aglycone tomatidine on HL-60 cells were investigated. Although previous studies have revealed that tomatidine was able to interact and form a complex with cell membrane-associated cholesterol (23,24), it was found that tomatidine had little effect on growth inhibition and apoptosis at the doses assessed. Other studies have demonstrated that glycoalkaloids caused membrane disruption leading to the release of peroxidase previously enclosed in lipid vesicles (33,34). It was observed that α-tomatine increased the fluorescence-measured membrane permeability of frog embryos while tomatidine had a weak effect (5). α-tomatine also decreased active transport via the activity of the sodium-potassium pump in frog skin, however, tomatidine had no effect on frog skin (5). These results, together with the present findings suggest that the glycone component is important for mediating the effects of α-tomatine on growth inhibition and apoptosis. In additional studies, it was investigated whether pre-treatment with the phorbol ester TPA may protect HL-60 cells from apoptosis induced by α-tomatine. Although the present study demonstrated that treatment with TPA resulted in a marked activation of NF-κB, pre-treatment with TPA did not protect HL-60 cells from α-tomatine-induced apoptosis. This result suggests that α-tomatine may also trigger pathways that are not associated with NF-κB to induce apoptosis in HL-60 cells.

The present study also demonstrated an *in vivo* effect of α-tomatine. Treatment of SCID mice with IP injection of α-tomatine three times a week significantly inhibited the growth of HL-60 cells *in vivo*. At the dose of 5 mg/kg body weight used in the present study, α-tomatine appeared to be non toxic as no body weight loss and no abnormalities in the major organs were observed in the animals. Previous studies revealed that α-tomatine was not toxic when consumed orally by rats (35–37). When administered intravenously, it had a median lethal dose value equal to 18 mg/kg body weight (35,36). Nishie *et al* (38) revealed that IP injection of α-tomatine (30–100 mg/kg) in rabbits produced neither fatalities nor abnormal ECG signals. Although the plasma concentrations of α-tomatine in these studies were not known, it is reasonable to assume that blood concentration of α-tomatine at micromolar level may be achievable without severe toxicity. Further studies are required to establish the plasma levels of α-tomatine in association with its inhibitory effect on leukemia and other types of cancer in suitable animal models.

In conclusion, the present study demonstrated that α-tomatine had a marked inhibitory effect on growth and a marked stimulatory effect on apoptosis in human myeloid leukemia HL-60 cells. α-tomatine also significantly inhibited the *in vivo* growth of HL-60 cells in the SCID mouse xenograft model. The present study suggests that cell membrane-associated cholesterol serves as a primary target for mediating the effect of α-tomatine in leukemia cells. The present study also indicates that the glycone component is critical for α-tomatine to convey signals for growth inhibition and apoptosis. The results from the present study indicate that α-tomatine may be a candidate for the development of novel anti-leukemia agents.
